# Post hoc estimation of a quantitative restriction spectrum imaging biomarker for prostate cancer detection using conventional MRI

**DOI:** 10.1002/acm2.70543

**Published:** 2026-03-19

**Authors:** Deondre D. Do, Christopher C. Conlin, Aditya Bagrodia, Matthew Cooperberg, Michael E. Hahn, Mukesh Harisinghani, Gary Hollenberg, Juan Javier‐Desloges, Sophia C. Kamran, Christopher J. Kane, Kang‐Lung Lee, Michael A. Liss, Daniel J. A. Margolis, Paul M. Murphy, Nabih Nakrour, Michael A. Ohliger, Thomas Osinski, Rebecca Rakow‐Penner, Mariluz Rojo Domingo, Amirali Salmasi, Ahmed S. Shabaik, Yuze Song, Shaun Trecarten, Natasha Wehrli, Eric P. Weinberg, Sean Woolen, Anders M. Dale, Tyler M. Seibert

**Affiliations:** ^1^ Department of Bioengineering University of California San Diego La Jolla California USA; ^2^ Department of Radiation Medicine University of California San Diego La Jolla California USA; ^3^ Department of Radiology University of California San Diego La Jolla California USA; ^4^ Department of Urology University of California San Diego La Jolla California USA; ^5^ Department of Urology University of California San Francisco San Francisco California USA; ^6^ Department of Radiology Massachusetts General Hospital Boston Massachusetts USA; ^7^ Department of Imaging Sciences University of Rochester Medical Center Rochester New York USA; ^8^ Department of Radiation Oncology Massachusetts General Hospital Boston Massachusetts USA; ^9^ Department of Radiology University of Cambridge Cambridge UK; ^10^ Department of Radiology Weill Cornell Medical College New York New York USA; ^11^ Department of Radiology and Biomedical Imaging University of California San Francisco San Francisco California USA; ^12^ Department of Urology University of Rochester Medical Center Rochester New York USA; ^13^ Department of Pathology University of California San Diego La Jolla California USA; ^14^ Department of Electrical and Computer Engineering University of California San Diego La Jolla California USA; ^15^ Department of Urology University of Texas Health Sciences Center San Antonio San Antonio Texas USA; ^16^ Department of Neurosciences University of California San Diego La Jolla California USA; ^17^ Halıcıoğlu Data Science Institute University of California San Diego La Jolla California USA; ^18^ Center for Multimodal Imaging and Genetics J. Craig Venter Institute La Jolla California USA

**Keywords:** diffusion weighted imaging, prostate cancer, radiation therapy, restriction spectrum imaging

## Abstract

**Background:**

Multiparametric MRI is useful for early detection of clinically significant prostate cancer (csPCa), but its standard apparent diffusion coefficient (ADC) has limited utility as a quantitative metric for automated, patient‐level detection of csPCa. Restriction spectrum imaging (RSI), an advanced diffusion technique, yields a quantitative biomarker (RSIrs) that improves csPCa detection. RSIrs is typically calculated from a dedicated multi‐*b*‐value acquisition. RSIrs estimated from conventional MRI has not been studied.

**Purpose:**

To evaluate the accuracy and validity of RSI metrics estimated post hoc from conventional diffusion‐weighted imaging (DWI) to serve as a viable surrogate for a dedicated RSI acquisition.

**Materials and Methods:**

We conducted a retrospective, multicenter study of patients with both a dedicated RSI acquisition and conventional DWI. We compared three different RSI restriction score (RSIrs) calculation methods: from the dedicated acquisition (RSIrs_dedicated_), from conventional DWI alone (RSIrs_post‐hoc_), and from a combination of conventional DWI with only the high *b*‐values from the RSI acquisition (RSIrs_combo_). We compared these methods for quantitative agreement and csPCa detection performance (area under the receiver operating characteristic [AUC, 95% confidence interval]) of maximum RSIrs (RSIrs_max_) in the prostate compared to that of minimum ADC (ADC).

**Results:**

Data from *n* = 1095 patients (16 centers) were analyzed. Post hoc RSIrs_max_ differed systematically from RSIrs_dedicated_ by a median of +156 (RSIrs_post‐hoc_) and −59 (RSIrs_combo_), respectively. AUCs for csPCa detection were 0.51 [0.47,0.54], 0.60 [0.57,0.64], 0.70 [0.67,0.74], and 0.77 [0.74,0.80] for ADC, RSIrs_post‐hoc_, RSIrs_combo_, and RSIrs_dedicated_, respectively.

**Conclusion:**

Even when estimated using conventional DWI, RSIrs is a superior quantitative biomarker to ADC for automated, patient‐level detection of csPCa. A dedicated RSI acquisition gives the best performance. A compromise would be to acquire high *b‐*values (1500 and 2500 s/mm^2^) to complement low *b‐*values (<1000 s/mm^2^) from conventional DWI.

## 1 INTRODUCTION

Multiparametric MRI (mpMRI) is useful for the detection and management of prostate cancer.^1^, ^2^, ^3^, ^4^, ^5^, ^6^ mpMRI includes diffusion‐weighted imaging (DWI) that permits calculation of a quantitative metric, the apparent diffusion coefficient (ADC). This metric is used to localize restricted water movement, a characteristic feature of hypercellular csPCa. Used in tandem with other image sequences in accordance with the Prostate Imaging Reporting & Data System (PI‐RADS), ADC maps help detect csPCa lesions on prostate MRI.^7^ However, the utility of ADC by itself as a quantitative metric is limited, in part because a lesion must first be identified by an experienced radiologist.

The model for ADC oversimplifies the complex, multistructural nature of water diffusion in human tissue.^8^, ^9^, ^10^ Restriction spectrum imaging (RSI) is a more sophisticated DWI technique that better accounts for tissue microstructure.^11^ The typical RSI framework models the diffusion MRI signal as a combination of exponential decays, corresponding to four distinct microcompartments: intracellular, extracellular, free diffusion (e.g., in the lumina of glands), and vascular flow. A quantitative biomarker derived from RSI—the RSI restriction score (RSIrs)—is useful for localizing csPCa. Previous work has demonstrated that RSIrs is superior to standard ADC for detecting csPCa on a patient‐level, defining tumor boundaries for radiotherapy targeting, and helping nonexperts identify csPCa on mpMRI.^9^, ^12^, ^13^

The RSI modeling framework can be applied to any conventional DWI dataset, provided enough nonzero *b*‐values were acquired to estimate the model parameters. However, it is unclear whether RSIrs can be accurately estimated from conventional DWI data used for PI‐RADS. Estimating RSI metrics with conventional DWI could be attractive for retrospective studies where only conventional DWI has been acquired. Additionally, some centers might want to implement RSI but prefer not to change their acquisition protocols.

We retrospectively applied the prostate RSI model to conventional DWI data in a cohort of patients who were scanned with both conventional DWI and a dedicated RSI sequence. This allowed for a direct, within‐patient comparison of post hoc RSIrs (RSIrs_post‐hoc_) to dedicated RSIrs (RSIrs_dedicated_). We also investigated RSIrs estimated from a combination of conventional DWI and RSI data (RSIrs_combo_) to determine if supplementing conventional DWI with additional *b*‐values could improve performance. We hypothesized that post hoc RSI would have some quantitative agreement with RSI and would remain superior to conventional ADC for fully automated, patient‐level detection of csPCa.

## 2 METHODS

### 2.1 Study population and data source

This retrospective analysis utilized a multicenter dataset drawn from six imaging centers. All data were collected under protocols approved by each center's respective Institutional Review Board. In accordance with these protocols, some data were used after written informed consent as part of prospective studies, while others were analyzed under a waiver of consent for the retrospective use of clinical data (at centers using RSI clinically).

The study cohort comprised men aged 18 years or older who underwent 3T prostate MRI with RSI between January 2016 and March 2024 for the evaluation of suspected or known csPCa. This included patients with no prior diagnosis (i.e., due to elevated PSA). In accordance with the International Society of Urological Pathology (ISUP) consensus, csPCa was defined as Grade Group ≥ 2 (Gleason score ≥ 3+4). Patient cases were considered non‐csPCa if they had histopathologic confirmation (benign or Grade Group 1) from a biopsy performed within 6 months of the MRI. For patients who—consistent with guidelines—did not undergo biopsy, non‐csPCa status was also assigned to cases with PI‐RADS 1–2 combined with a low PSA density (PSAD ≤ 0.15 ng/mL^2^). This definition introduces the possibility of a small number of cases with csPCa incorrectly assumed to be non‐csPCa, which could artificially diminish the measured performance of RSI or other metrics. This risk is small, and the approach confers better statistical power to measure overall cancer discrimination. Patients were excluded if they had received treatment for prostate cancer prior to their MRI or if they had metallic implants that could cause significant imaging artifacts. Patients must have had both an acquisition of a conventional DWI sequence (including a full field‐of‐view [FOV] and reduced‐FOV [FOCUS] acquisition) and a separate, dedicated RSI sequence during the same imaging session.

### 2.2 RSI data acquisition, processing, and modeling

All DWI data (conventional and RSI) underwent a standardized preprocessing pipeline that included correction for background noise, geometric distortion correction, gradient nonlinearities, *B_0_* inhomogeneities, and eddy currents.^8^, ^14^ To account for varying FOV strategies across sites, reduced‐FOV (FOCUS) acquisitions were prioritized for spatial orientation due to minimized geometric distortion compared to full‐FOV acquisitions. Therefore, all conventional DWI data were resampled into the FOCUS imaging space, followed by rigid‐body registration with mutual information performed using the *b *= 0 (s/mm^2^) acquisitions of each available scan to correct for minor patient motion that may have occurred between the acquisitions. All patients included in this study had both full‐FOV and FOCUS acquisitions available. Automated whole‐prostate segmentations were generated for each patient based on the T2‐weighted images using a validated deep‐learning‐based tool.^15^ For the primary patient‐level analysis, RSIrs and ADC metrics were extracted from these whole‐prostate segmentations. Manual lesion‐level contours were reserved strictly for the secondary validation subset to evaluate spatial concordance between RSIrs metrics and histopathology. To account for signal intensity differences between the two scans, a scaling factor was calculated from the ratio of the median signal intensities within the prostate mask on the *b *= 0 (s/mm^2^) images of the available data. The final post hoc DWI volume is then processed using the multicompartmental RSI model. For conventional DWI with inadequate (<4) *b*‐values to model the four‐compartment RSI model parameters, a simplified three‐compartment RSI model framework was used instead to prevent unstable model solutions.^11^ No parameters were re‐optimized for the current multicenter dataset to ensure the evaluation of model generalizability and avoid overfitting. The set of compartmental diffusivities differed between the three‐ and four‐compartment frameworks (see Table S2) to maintain optimal fitting of the diffusion decay curve given the available data. Specifically, the restricted intracellular component varied (D_1_ = 5.2 × 10^−4^ vs D_1_ = 8.7 × 10^−4^ mm^2^/s) for the four‐ versus three‐compartment RSI model. Attempting to fit a four‐compartment RSI model with insufficient data points leads to unstable model solutions not representative of diffusion in the tissue microcompartments. The RSIrs biomarker is then derived from the intracellular signal component (RSI‐C_1_), normalized by the median DWI signal at *b *= 0 (s/mm^2^) of the prostate to reduce scanner‐dependent signal variation (Equation 1). For protocols without a *b* = 0 (s/mm^2^) acquisition in their conventional DWI scans, normalization was done with the median DWI signal of the patient's lowest *b*‐value acquisition.

(1) $$S\left(b\right)=\sum_{i=1}^{4}C_{i}e^{-bD_{i}}\to RSIrs=\frac{C_{1}}{mb0}$$

Equation (1): Formula to compute RSIrs where S(*b*) represents the RSI signal. *C_i_* denotes the RSI compartment signal contribution, *D_i_* is the compartmental diffusion coefficient, and *mb*0 is the median DWI signal in the prostate (*x* ∈ {0, 50})

For this study, four distinct quantitative RSIrs maps were generated for each patient:

1. RSIrs_dedicated_ (reference standard): Calculated with diffusion data from the complete dedicated multi‐*b*‐value RSI dataset.
2. RSIrs_post‐hoc_: Estimated by applying the RSI model using only the *b*‐values available from the patient's conventional DWI scan (including a full‐FOV [Full FOV] and smaller FOV focused on the prostate [FOCUS])
3. RSIrs_combo_: Estimated from a combined dataset comprising of Full FOV data from the conventional DWI with low *b*‐values (*b *≤ 1400 s/mm^2^ from the Full FOV scan) supplemented with a subset of high *b*‐values (>1400 s/mm^2^) from the dedicated RSI scan.
4. ADC: Apparent diffusion coefficient maps were calculated in standard fashion using *b*‐values <1000 s/mm^2^. To establish the baseline for a simple, automated quantitative biomarker, the minimum ADC value across the entire prostate gland was recorded for each patient. We utilized all available *b*‐values (s/mm^2^) from the dedicated RSI acquisition to establish the reference standard ADC maps. We used the dedicated RSI scan's low *b*‐values rather than the conventional DWI's to eliminate any potential registration errors affecting ADC calculations. This whole‐gland metric was chosen to test ADC's capability of providing a single patient‐level quantitative biomarker for detecting csPCa, with the understanding that it is susceptible to known confounders like benign prostatic tissue. It is not how ADC is typically used clinically (which would require subjective identification of suspicious lesions by experienced radiologists). Rather, it is an analogous comparator to RSIrs as a quantitative biomarker for automated, patient‐level detection.

All post‐processing was performed using custom scripts developed in MATLAB (MathWorks, Natick, MA).

### 2.3 Comparative analysis

To isolate the diagnostic performance of the ADC and RSI signal models, we utilized a fully automated, reader‐independent comparison, consistent with previous studies.^13^, ^16^, ^17^, ^18^, ^19^, ^20^ While minimum ADC is not used in clinical practice, it serves as the closest analog to maximum RSIrs, leveraging the only quantitative metric in widespread clinical use today. Typical practice is for a radiologist to identify a suspicious lesion and then to obtain the mean ADC within that lesion, but this process is inherently subjective and depends on the expertise of the given radiologist. By comparing RSIrs​ and ADC directly, without the use of expert‐defined lesions, we can evaluate the utility of each for potential use as an objective, automated, quantitative imaging biomarker for csPCa.

### 2.4 Statistical analysis

All statistical analyses were performed in Python (v3.8) using the scipy, pandas, and scikit‐learn libraries. As the maximum RSIrs is typically used for detecting csPCa, we use this metric as our standard for comparison of post hoc estimations of RSIrs to dedicated RSIrs. Our evaluation focused on two primary outcomes:

**Quantitative bias and agreement**: Bland–Altman analyses were performed to find the maximum RSIrs difference (bias) and 95% limits of agreement (LoA) between the post hoc RSIrs estimation methods and the dedicated RSI reference standard. We directly compared the post hoc methods to each other by calculating the error in maximum RSIrs of each method to the reference standard for each patient. The statistical significance of the median bias was assessed using a two‐sided Wilcoxon signed‐rank test, with significance set at alpha 0.05.

**csPCa detection performance**: To compare the automated/quantitative, patient‐level csPCa detection potential of each metric, we generated receiver operating characteristic (ROC) curves and calculated the area under the curve (AUC) for each of the four imaging metrics. Ninety‐five percent confidence intervals (95% CIs) were generated using 10 000 patient bootstraps for each metric.

We conducted the above analyses stratifying by protocol, model framework (three vs. four compartment), and in a subset of patients with PI‐RADS lesions contoured on MRI and tumors contoured on whole‐mount histopathology by a subspecialist expert radiologist and pathologist, respectively, to investigate the performance of post hoc RSIrs in different patient subsets. Co‐registration of whole‐mount histopathology to MRI was done using the previously validated RAPSODI software.^21^

## 3 RESULTS

**FIGURE 1:**
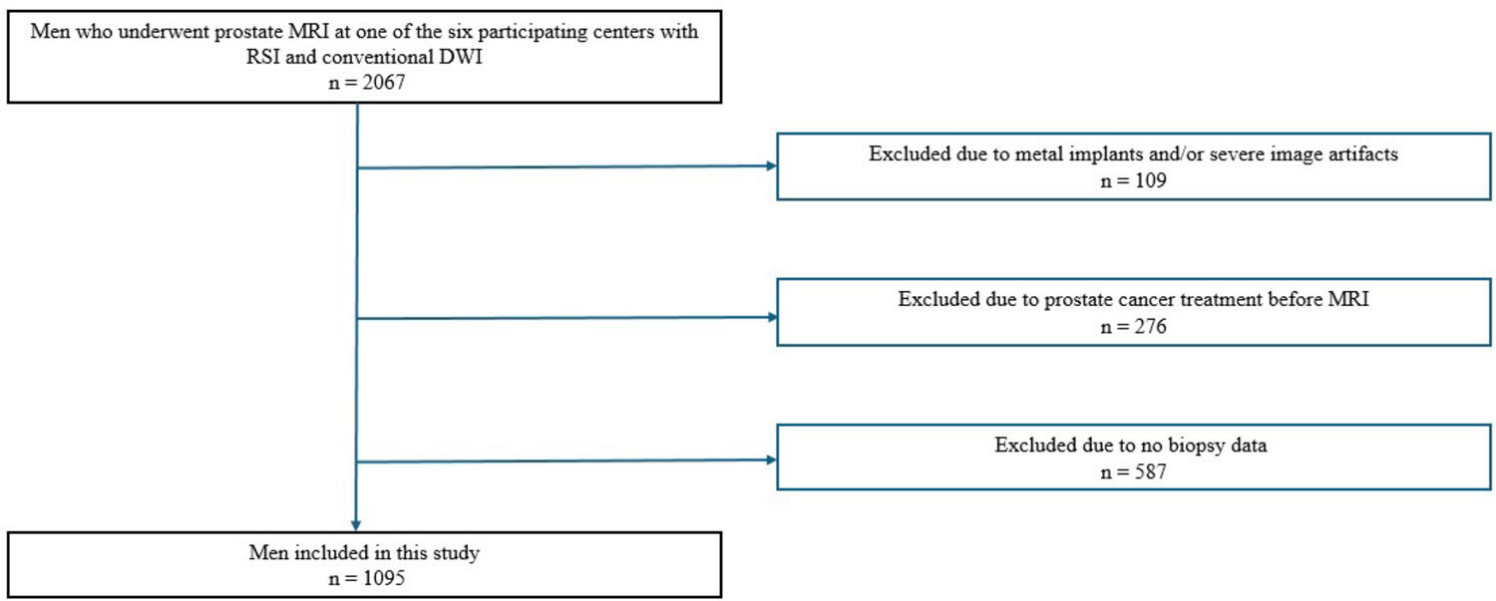
Patient cohort flowchart. Showing how the patient cohort was constructed for this study.

One thousand and ninety‐five patients were eligible for this study (Figure 1 and Table 1). MRI data were gathered from 16 MRI scanners (three unique models) from two vendors (GE Healthcare, Waukesha, WI, USA; SIEMENS Healthineers, Erlangen, Germany) and 12 unique DWI protocols (six conventional and six dedicated RSI). Detailed parameters can be found in Table S1.

**TABLE 1:** Patient characteristics.

Institutions	
Institution 1	216
Institution 2	501
Institution 3	86
Institution 4	194
Institution 5	56
Institution 6	42
Age, median (IQR), year	70 (64–75)
PSA, median (IQR), ng/Ml	6.40 (4.70–9.20)
Prostate volume, median (IQR), mL	56 (42–78)
PSA density, median (IQR), ng/mL^2^	0.10 (0.07–0.17)
**Biopsy status**	
Received biopsy before MRI scan	477
Biopsy‐naïve at time of MRI scan	735
**Biopsy pathology**	
Systematic biopsy only	184
Targeted biopsy only	131
Systematic and targeted biopsy	433
Whole‐mount histopathology in patients who received radical prostatectomy	64
**Highest MRI PI‐RADS (v2.1) lesions**	
PI‐RADS score 1	346
PI‐RADS score 2	37
PI‐RADS score 3	180
PI‐RADS score 4	273
PI‐RADS score 5	255
No PI‐RADS scores (research‐only scans)	4
**Total MRI PI‐RADS (v2.1) lesions**	
Total number of PI‐RADS lesions	1091
Total negative (1, 2) PI‐RADS lesions	382
Total positive (3, 4, 5) PI‐RADS lesions	709
**Gleason grade group pathology**	
Benign	219
Grade Group 1	198
Grade Group 2	214
Grade Group 3	115
Grade Group 4	45
Grade Group 5	54
**Self‐reported race and ethnicity**	
White, Hispanic	56
White, non‐Hispanic	706
White, ethnicity other/unknown	47
Asian	71
Black or African American	76
American Indian/Alaska Native	2
Native Hawaiian or other Pacific Islander	5
Other/Unknown	132

Abbreviations: IQR, interquartile range; PI‐RADS, Prostate Imaging Reporting and Data System; PSA, prostate‐specific antigen; PSAD, PSA density.

**FIGURE 2:**
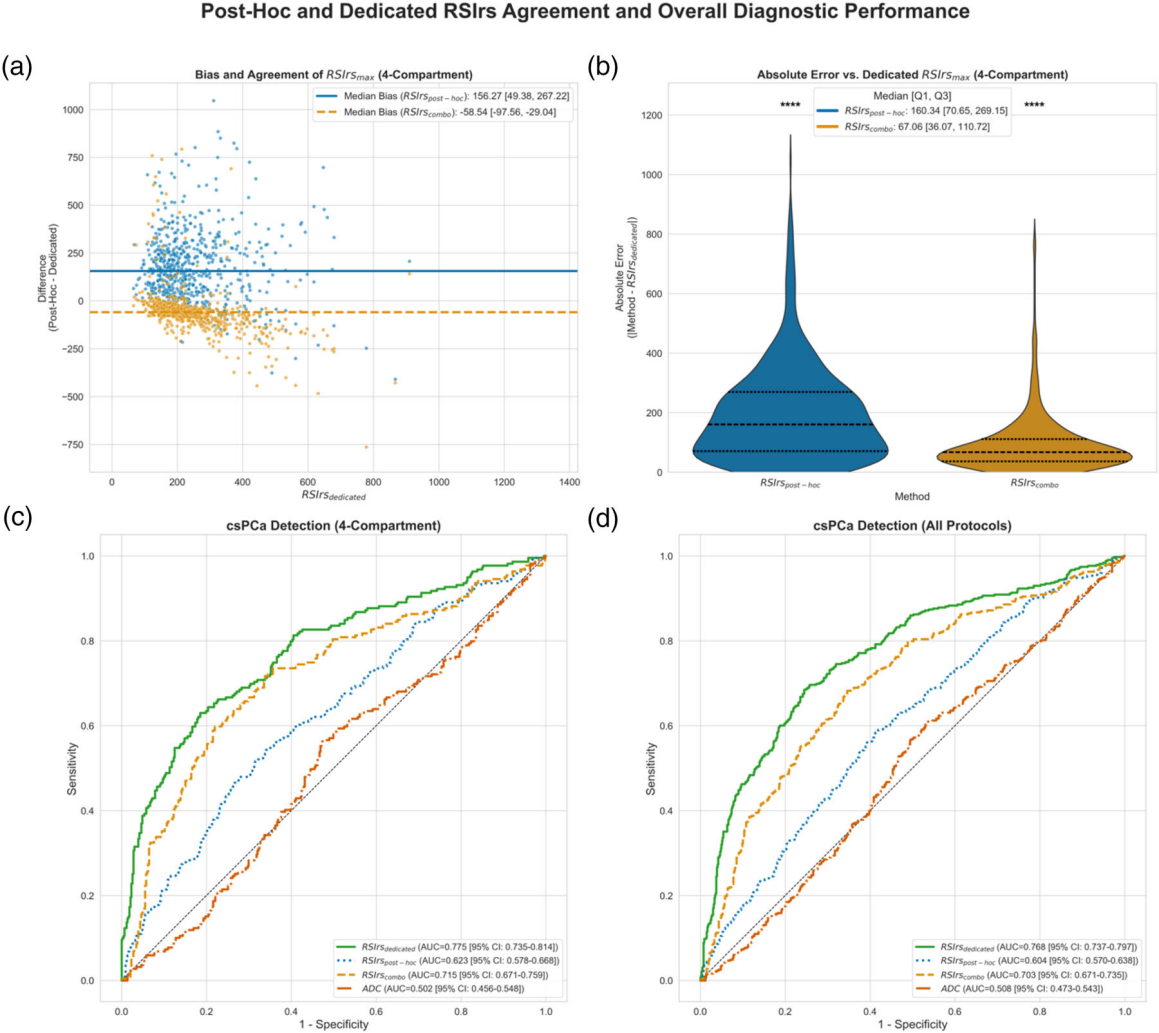
Quantitative comparison of post hoc RSI to dedicated RSI. Plots (a) and (b) show Bland–Altman and violin plots showing the bias, agreement, and absolute error between post hoc RSI methods and dedicated RSI using the four‐compartment RSI model, respectively. Each dot represents an individual patient and their bias of post hoc to dedicated RSIrs; each patient has two dots, one for each post hoc RSIrs. Plots (c) and (d) display receiver operating characteristic curves for the detection of clinically significant prostate cancer (csPCa) for four‐compartment model and all data (a three‐compartment RSI model was used if only three nonzero *b*‐values were available) of post hoc RSI, dedicated RSI, and the apparent diffusion coefficient (ADC). The plots showcase the hierarchy of performance: dedicated RSI > combination of conventional diffusion weighted imaging (DWI) and RSI > conventional DWI alone > ADC. Based on significance level of Wilcoxon signed‐rank test between dedicated RSI and post hoc RSI methods, a significance indicator above each respective violin plot is shown (*p* < 0.0001: ****, *p* < 0.001: ***, *p* < 0.01: **, *p* < 0.05: *, not significantly different: “ns”).

Using conventional DWI data adequate to fit the four‐compartment model (≥4 nonzero *b‐*values, *n* = 651 patients), RSIrs_post‐hoc_ tended to overestimate RSIrs values (median bias: +156.3; IQR: [49.4, 267.2]; mean [95% LoA]: +173.5 [−182.4, 529.5]; *p* < 0.001). A similar pattern was observed in the cohort of protocols (*n* = 444) using the three‐compartment model (median: +100.7; IQR: [9.8, 193.8]; mean [95% LoA]: +109.9 [−199.0, 418.8]; *p* < 0.001) (Figure 2a). The absolute average error was reduced with the RSIrs_combo_ method (median: −58.5; IQR: [−97.6, −29.0]; mean [95% LoA]: −55.5 [−308.4, 197.5]; *p* < 0.001) (Figure 2b). Analysis of subgroups based on center and acquisition protocol revealed that this improvement was driven mostly by datasets lacking a high *b*‐value in the conventional DWI data. For example, in data from Institution 6 (conventional DWI *b*‐values: 0, 600, 1000 s/mm^2^), RSIrs_post‐hoc_ demonstrated a very large overestimation bias (median: +188.7; IQR: [123.5, 257.9]; mean [95% LoA]: +168.3 [−200.5, 537.1]; *p* < 0.001), but RSIrs_combo_ greatly reduced the average error (median: −45.6; [−65.8, −13.9]; mean [95% LoA]: −65.6 [−274.0, +142.9]; *p* < 0.001) (Figure S1b). On the other hand, in data from Institution 2 (conventional DWI *b*‐values: 50, 1000, 1400 s/mm^2^), RSIrs_post‐hoc_ showed no significant systematic bias from the reference (median: +3.9; IQR: [−33.1, 48.4]; mean [95% LoA]: +0.7 [−159.3, 160.6]; *p* = 0.445), and RSIrs_combo_ modestly worsened the average estimate (median: −44.5; IQR: [−81.5, −14.4]; mean [95% LoA]: −46.6 [−173.0, +79.7]; *p* < 0.001) (Figure S1d).

**FIGURE 3:**
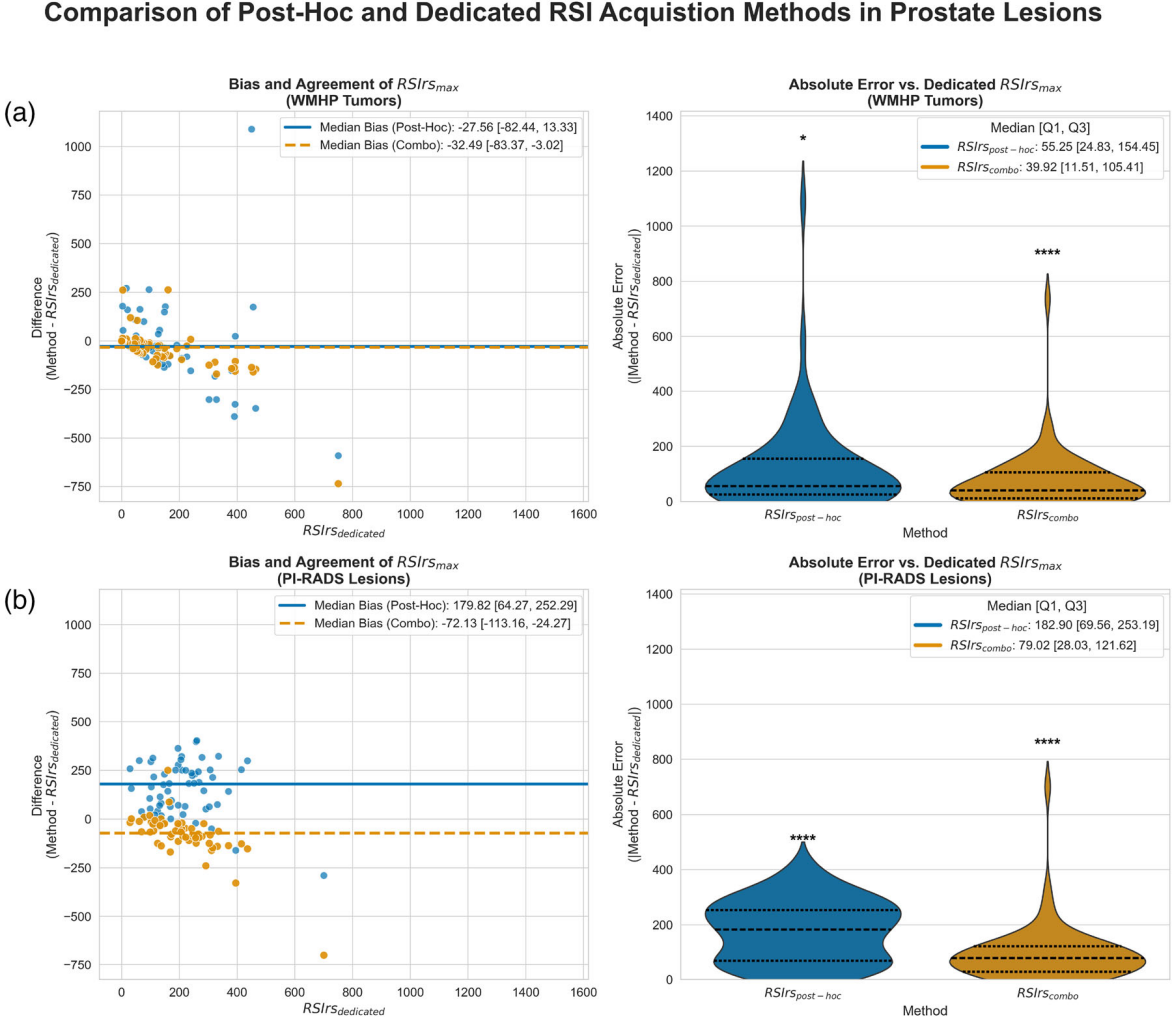
Quantitative comparison of post hoc RSI to dedicated RSI in expert‐defined tumors/lesions. Plots (a) and (b) showcase the bias, agreement, and absolute error between post hoc and dedicated RSI metrics within prostate tumors/lesions contoured by clinical experts. In both plots, the left graph shows a Bland–Altman analysis, and the right graph shows two violin plots comparing RSIrs post hoc and RSIrs combo to RSIrs dedicated. Each dot represents an individual patient and their bias of post hoc to dedicated RSIrs; each patient has two dots, one for each post hoc RSIrs. Agreement with RSIrs dedicated was high for post hoc methods in WMHP tumors, but there is a noticeable discrepancy in PI‐RADS lesions, overall (which may or may not be cancer). RSIrs combo improves agreement in both patient subsets overall. Based on significance level of Wilcoxon signed‐rank test between dedicated RSI and post hoc RSI methods, a significance indicator above each respective violin plot is shown (*p *< 0.0001: ****, *p* < 0.001: ***, *p* < 0.01: **, *p* < 0.05: *, not significantly different: “ns”).

Lesion‐level evaluation of post hoc RSIrs estimates was feasible in a subset of patients with contoured PI‐RADS lesions and corresponding pathology diagnosis from whole‐mount histopathology (Figure 3). In PI‐RADS defined lesions (*n* = 58), RSIrs_post‐hoc_ bias was (median: +179.8 IQR: [64.3, 252.3]; mean [95% LoA]: +159.1 [−106.0 to 424.1]; *p* < 0.001) and RSIrs_combo_ bias was (median: −72.1; IQR: [−113.2, −24.3]; mean [95% LoA]: −79.9 [−302.9 to 143.0]; *p* < 0.001). In tumors confirmed on whole‐mount histopathology (*n* = 64), RSIrs_post‐hoc_ bias was (median: −27.6; IQR: [−82.4, 13.3]; mean [95% LoA]: −21.9 [−422.0 to 378.2]; *p* = 0.036), while RSIrs_combo_ bias was (median: −32.5; IQR: [−83.4, −3.0]; mean [95% LoA]: −46.8 [−277.9 to 184.4]; *p* < 0.001).

Quantitative csPCa detection was significantly better with dedicated RSI acquisition than with post hoc methods, though all RSIrs estimates had better performance than ADC (Figure 2d). RSIrs_dedicated_ achieved the highest accuracy (AUC: 0.77; 95% CI: [0.74, 0.80]). RSIrs_combo_ performed second best (AUC: 0.70; 95% CI: [0.67, 0.74]), RSIrs_post‐hoc_ third (AUC: 0.60; 95% CI: [0.57, 0.64]), and ADC last (AUC: 0.51; 95% CI: [0.47, 0.54]).

We repeated the primary analyses with a three‐compartment model for those subsets of data where the conventional DWI included insufficient *b*‐values to permit fitting a four‐compartment model. The patterns of results were similar to those in the four‐compartment model analyses (see Figure S2). We also repeated the primary analyses estimating post hoc RSIrs in a patient subset with only Full FOV (RSIrs_FullFOV_) or only FOCUS (RSIrs_Focus_) data, respectively (Figure S3); we found that RSIrs_Focus_ systematically had better csPCa detection performance than other post hoc estimation methods (RSIrs_post‐hoc_ and RSIrs_FullFOV_) but still fell short in performance compared to RSIrs_combo_.

## 4 DISCUSSION

In data from six imaging centers, we found that post hoc estimation of RSIrs is feasible and that post hoc RSIrs is still superior to ADC as a quantitative metric for prostate/patient‐level automated csPCa detection. Post hoc RSIrs can be used in retrospective analysis of datasets that did not have a dedicated RSI acquisition or as a temporary solution during the early stages of implementing dedicated RSI. However, post hoc RSI methods are not a complete substitute for a dedicated RSI acquisition. Estimation of RSIrs is best when a high *b*‐value (>1000 s/mm^2^) is acquired. These results can also inform efforts to balance constraints on acquisition protocols. Where feasible, a dedicated RSI acquisition is advised to optimize quantitative results. Given that ADC and average *b‐*value maps can be calculated from the DWI data in a multi‐*b‐*value RSI acquisition, if only one DWI acquisition can be performed, a dedicated RSI protocol can yield both conventional and post hoc RSIrs maps and metrics. If a dedicated RSI acquisition is not feasible, RSIrs may still be estimated.

The robust performance of RSIrs across this multi‐institutional dataset suggests that the fixed compartmental diffusivities (*D_i_*) are stable for estimating prostate tissue diffusion. Because the restricted and hindered diffusion compartments (C_1_ and C_2_) differ by nearly an order of magnitude in their diffusivity, the performance of RSIrs for detecting csPCa is unaffected by the presence of minor gradient calibration offsets or TE variations. While these hardware factors may introduce a systematic shift in absolute quantitative value, supporting the need for protocol‐specific calibration, they do not degrade the capability of RSIrs to detect csPCa. Results that agree with previous work.^16^

**FIGURE 4:**
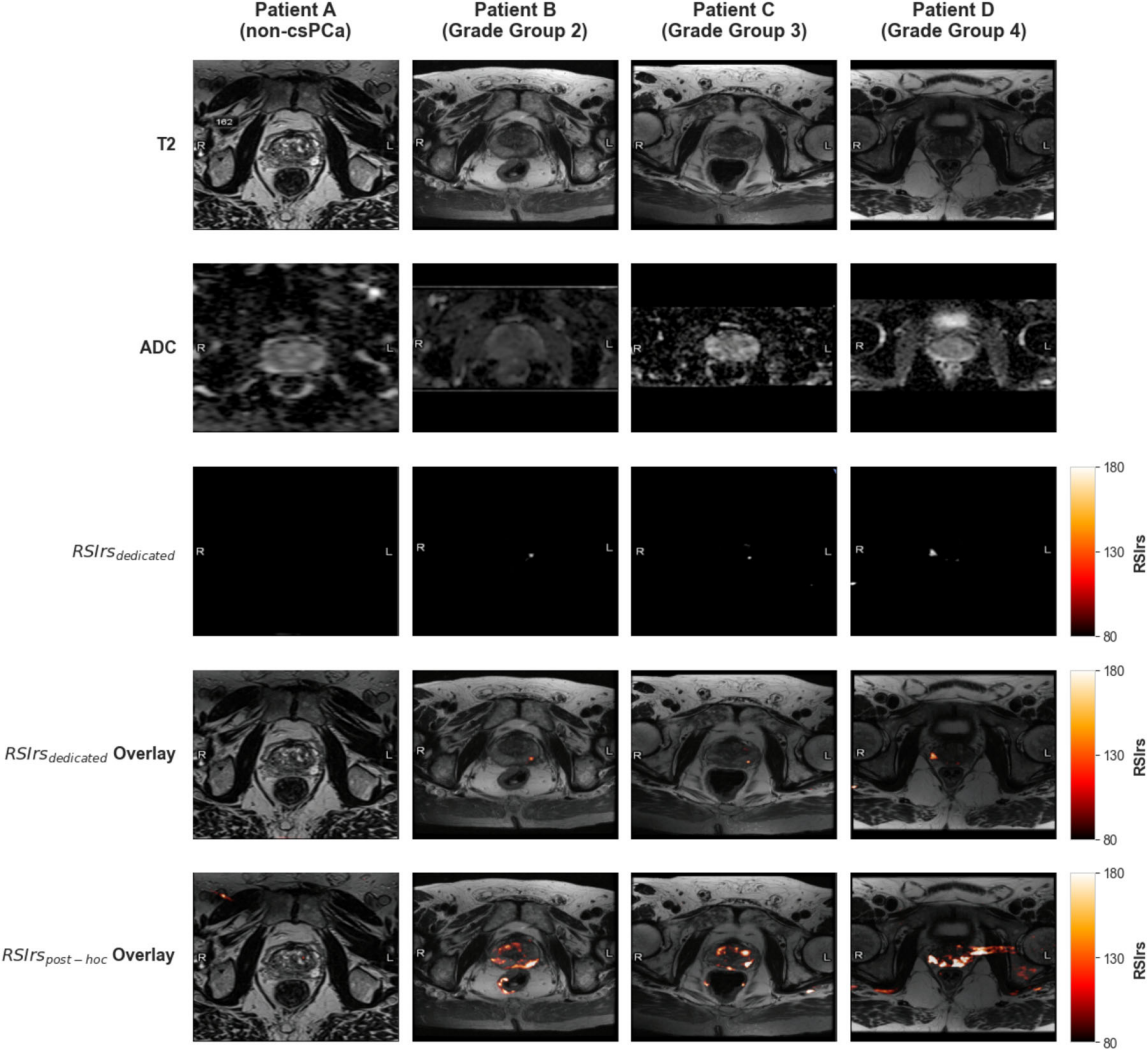
Examples of quantitative biomarker maps. Four representative patients (A–D). Patient A had no csPCa while Patients B–D had increasingly aggressive csPCa (B: Grade Group (GG) 2, C: GG3, D: GG4) and underwent radical prostatectomy. The corresponding slice on MRI is displayed in subsequent rows T2‐weighted imaging, apparent diffusion coefficient (ADC), dedicated RSIrs, and RSIrs (two versions) overlaid on T2. The color bar indicates the signal intensity windows used for all RSIrs maps. All patients had a four‐compartment post hoc RSIrs; a high *b*‐value was available from conventional DWI data for Patients B and C. Overall, RSIrsdedicated has the greatest agreement with location of csPCa tissue on RSI.

While the quantitative performance of post hoc RSI was superior to ADC, it is worth noting that the post hoc approach introduces systematic bias/error in the quantitative values. Furthermore, while the use of three‐compartment RSI modeling introduces a potential source of variability, as this approach is not intended to be the most optimal solution for calculating RSIrs, our sensitivity analysis (Figure S2) demonstrates that the diagnostic utility of RSIrs is largely preserved across both models. This stability arises because the restricted signal component (C_1_) is mathematically distinct from the fast diffusion compartments (C_3_ and C_4_). The primary challenge in post hoc estimation is the lack of high *b*‐values to suppress the extracellular hindered (C_2_) signal, rather than the partitioning of the fast‐diffusion components that are combined in the three‐compartment model. Consequently, the RSI framework remains superior to mono‐exponential ADC even when the model order is constrained by clinical protocol limitations. Clinically relevant thresholds like those described for dedicated RSIrs ^19^ likely need to be recalibrated for post hoc RSIrs (Figure 4). This calibration would need to be performed for each acquisition protocol, as conventional DWI protocols can vary markedly across centers. Conversely, previous work has shown that dedicated RSIrs is relatively robust to variation in center, protocol, scanner, and patient demographics.^16^ The strong csPCa detection performance of RSIrs_combo_ is encouraging. It demonstrates that the performance of post hoc RSI methods can be substantially improved by incorporating even just one high *b*‐value into the standard clinical protocol. Overall, this study provides two alternative strategies to retrospectively analyze conventional DWI data and assist centers who are hesitant to alter their standard protocols to implement RSI by applying post hoc RSI accordingly based on the availability of high *b*‐value data. A simple addition of one high *b*‐value acquisition (i.e., *b* = 1500 s/mm^2^) would only minimally increase protocol acquisition time to gain the benefits of RSIrs.

Consistent with prior studies, our findings demonstrate that whole‐gland minimum ADC is a poor marker for the presence of csPCa and is significantly outperformed by RSIrs.^19^, ^22^ It is important to distinguish between this illustrative quantitative benchmark and standard clinical use of ADC. According to PI‐RADS v2.1, radiologists integrate focal signal intensities on ADC maps with morphology on T_2_‐weighted imaging to detect csPCa. An experienced radiologist first identifies a suspicious lesion and then semiquantitatively evaluates the mean ADC within the lesion relative to (subjectively defined) normal‐appearing prostate tissue. The reliance on expert readers introduces interobserver variability that is the motivation for the pursuit of a quantitative imaging biomarker. The whole‐gland approach for RSIrs demonstrates its potential as a quantitative biomarker that has utility without first identifying a lesion. As ADC is the only quantitative DWI metric in widespread clinical use, we apply an analogous methodology (i.e., minimum ADC in the prostate) to illustrate the relative potential utility of the novel RSIrs and the standard ADC for objective, quantitative prediction of biopsy outcomes. We found that even post hoc RSIrs significantly distinguishes prostates that contain csPCa from those that do not. Our results suggest that post hoc RSIrs could be a viable, albeit suboptimal, option for centers looking to migrate to a dedicated RSIrs protocol and for retrospective validation studies that only have legacy DWI data available.

There are several factors to consider regarding the implementation of RSI into clinical workflow. The transition of RSI from a research framework to a clinical tool involves overcoming operational barriers in scan time and workflow integration. Dedicated RSI protocols, while providing optimal csPCa detection performance, may increase acquisition time by a few minutes compared to conventional DWI protocols. The RSIrs_combo_ approach presented here offers a pragmatic compromise, requiring only an approximately 90‐s additional scan time for a single high‐*b*‐value shell to a conventional DWI protocol, albeit as a compromise compared to an only slightly longer dedicated RSI acquisition. Currently, RSIrs maps require automated offline processing to calculate the RSIrs biomarker; however, the use of Docker‐based pipelines ensures seamless integration with PACS, allowing for the delivery of color‐overlay RSIrs heatmaps directly to the radiologist's workstation with minimal effort after initial setup. Previous work^9^ suggests that this improves tumor conspicuity and reduces the cognitive load on the reader by highlighting the tumor location on MRI. Work is currently being conducted studying the impact of RSIrs maps on radiologist performance in a prospective, multicenter study.^23^ CPT codes exist for 3D post‐processing, which may facilitate reimbursement for the (modest) additional acquisition time. A commercial version of RSI is FDA cleared and in routine use at some centers in the United States, suggesting feasibility, though broad reimbursement implications warrant further study as more centers adopt quantitative biomarkers.

We acknowledge that a portion of our cohort had DWI acquisitions that did not adhere to the PI‐RADS v2.1 recommendation to include *b* > 1400 s/mm^2^ and instead used a synthetic high *b‐*value, as permitted by the guidelines. This further increases the already inherent variability in multi‐institutional data. Rather than a limitation, we view our inclusion of these data as a rigorous test of the generalizability of the RSI framework across different vendor platforms and clinical environments. Our results demonstrate that even with suboptimal data, RSIrs remains a superior quantitative biomarker to ADC, highlighting its potential for retrospective research and longitudinal oncology studies.

A limitation of this study is its reliance on PI‐RADS interpretation and biopsy results as the ground truth, where interpretation heavily relies on the reader expertise. Biopsy techniques are also prone to error. On the other hand, whole‐mount histopathology is only available for patients who are first diagnosed with csPCa, are candidates for surgery, and who elect to undergo radical prostatectomy. Thus, biopsy results, despite their imperfection, are the relevant clinical gold standard. This study is also limited by a lack of central radiology or pathology review. For some protocols, we note that a *b* = 0 acquisition was not available, so the lowest available *b*‐value was used to estimate RSIrs. The discrepancies in echo time​ between conventional and dedicated sequences introduce varying degrees of compartment‐specific T_2​_‐weighting to the diffusion signal. This, along with differing repetition time and other protocol‐driven variations, may contribute to the systematic overestimation bias observed in post hoc RSI estimation. Finally, even data from seven centers will not represent all possible conventional DWI protocols.

A technical limitation of this multicenter study is the lack of *b* = 0 s/mm^2^ acquisitions in several protocols, requiring normalization of the RSIrs biomarker using other low *b*‐value acquisitions (*b* = 50 s/mm^2^). The attenuation of the diffusion signal at *b *= 50 s/mm^2^ results in a lower normalization denominator, leading to a systematic overestimation of absolute RSIrs values. While this shift suggests the need for protocol‐specific calibration of diagnostic thresholds, it does not degrade csPCa discrimination. As demonstrated by the high AUCs across heterogeneous sites, the relative contrast in RSIrs between csPCa and benign tissue is preserved regardless of any shifts due to this difference in normalization.

## 5 CONCLUSION

RSIrs estimated post hoc from conventional DWI offers a significant improvement over standard ADC as a patient‐level quantitative biomarker of csPCa. RSIrs calculated from a dedicated, multi‐*b*‐value acquisition performs best. Post hoc RSIrs can be used in retrospective analyses where dedicated RSI data are not available and could also be implemented clinically at centers with heavy constraints on modifying their DWI protocols.

## AUTHOR CONTRIBUTIONS

**Deondre D. Do**: Conceptualization; methodology; software; validation; formal analysis; investigation; data curation; writing—original draft; writing—review and editing; visualization. **Christopher C. Conlin**: Software; writing—review and editing. **Aditya Bagrodia**: Data curation; writing—review and editing. **Matthew Cooperberg**: Data curation; writing—review and editing. **Michael E. Hahn**: Data curation, writing—review and editing. **Mukesh Harisinghani**: Data curation; writing—review and editing. **Gary Hollenberg**: Data curation; writing—review and editing. **Juan Javier‐Desloges**: Data curation; writing—review and editing. **Sophia C. Kamran**: Data curation; writing—review and editing. **Christopher J. Kane**: Data curation; writing—review and editing. **Kang‐Lung Lee**: Data curation; writing—review and editing. **Michael A. Liss**: Data curation; writing—review and editing. **Daniel J. A. Margolis**: Data curation; writing—review and editing. **Paul M. Murphy**: Data curation; writing—review and editing. **Nabih Nakrour**: Data curation; writing—review and editing. **Michael A. Ohliger**: Data curation; writing—review and editing. **Thomas Osinski**: Data curation; writing—review and editing. **Rebecca Rakow‐Penner**: Data curation; writing—review and editing. **Mariluz Rojo Domingo**: Data curation; writing—review and editing. **Amirali Salmasi**: Data curation; writing—review and editing. **Ahmed S. Shabaik**: Data curation; writing—review and editing. **Yuze Song**: Data curation; writing—review and editing. **Shaun Trecarten**: Data curation; writing—review and editing. **Natasha Wehrli**: Data curation; writing—review and editing. **Eric P. Weinberg**: Data curation; writing—review and editing. **Sean Woolen**: Data curation; writing—review and editing. **Anders M. Dale**: Data curation; writing—review and editing. **Tyler M. Seibert**: Conceptualization; methodology; software; validation; formal analysis; investigation; resources; data curation; writing—original draft; writing—review and editing; visualization; supervision; project administration; funding acquisition.

## CONFLICT OF INTEREST STATEMENT

Tyler M. Seibert reports honoraria from CorTechs Labs, Varian Medical Systems, WebMD, GE Healthcare, and Janssen; has an equity interest in CorTechs Labs, Inc, and serves on its scientific advisory board; and has received in‐kind research support from GE Healthcare via a research agreement with the University of California, San Diego. These companies might potentially benefit from the research results. The terms of these arrangements have been reviewed and approved by the University of California, San Diego in accordance with its conflict‐of‐interest policies. Anders M. Dale is a founder of and holds equity in CorTechs Labs, Inc, and serves on its scientific advisory board; he also is a member of the scientific advisory board of Human Longevity, Inc, and receives funding through research agreements with GE Healthcare. Health Research reported in this publication was supported by the National Cancer Institute of the National Institutes of Health under Award Number R01CA279667. The content is solely the responsibility of the authors and does not necessarily represent the official views of the National Institutes of Health.

## ETHICS STATEMENT

Participating institutions received approval from their respective institutional review boards (IRBs). Prospectively gathered data from Institution 3 were collected with written informed consent from participants, while the remaining institutions obtained a consent waiver from their IRBs for retrospective use of clinical records.

## Supporting information




**Supporting file 1**: acm270543‐sup‐0001‐SuppMat.docx

## Data Availability

All data and code used in this study are available upon reasonable request. Please send a request to the corresponding author Dr. Tyler Seibert at tseibert@health.ucsd.edu or the primary author Deondre Do at d2do@health.ucsd.edu.
